# Using angiogenic factors and their soluble receptors to predict organ dysfunction in patients with disseminated intravascular coagulation associated with severe trauma

**DOI:** 10.1186/cc11309

**Published:** 2012-04-20

**Authors:** Takeshi Wada, Subrina Jesmin, Satoshi Gando, Sayeeda N Sultana, Sohel Zaedi, Hiroyuki Yokota

**Affiliations:** 1 Division of Acute and Critical Care Medicine, Department of Anesthesiology and Critical Care Medicine, Hokkaido University Graduate School of Medicine, N17W5, Kita-ku, Sapporo 060-8638, Japan; 2 Division of Gene Therapeutics, Research Institute, National Center for Global Health and Medicine, 1-21-1 Toyama Shinjuku-ku, Tokyo 162-8655, Japan; 3 Health and Diseases Research Center for Rural Peoples (HDRCRP), 14/15, 1st floor, Probal Housing Ltd., Shekertak (Adjacent to Shekertak Road 1), Mohammadpur, Dhaka 1207. Bangladesh; 4 Department of Emergency and Critical Care Medicine, Nippon Medical School, 1-1-5 Sendagi Bunkyo-ku, Tokyo 113-8603, Japan

## Abstract

**Introduction:**

Disseminated intravascular coagulation (DIC) is characterized by the concomitant activation of coagulofibrinolytic disorders and systemic inflammation associated with endothelial dysfunction-induced microvascular permeability. Angiogenic factors, including vascular endothelial growth factor (VEGF), angiopoietin (Ang), and their receptors, play crucial roles in angiogenesis and microvascular permeability. The aim of the study was to assess the relationship between angiogenic factors, their soluble receptors and organ dysfunction associated with DIC after severe trauma.

**Materials and methods:**

A total of 57 patients with severe trauma were divided into two subgroups; 30 DIC patients and 27 non-DIC patients. The DIC was diagnosed based on the Japanese Association for Acute Medicine (JAAM) DIC and the International Society on Thrombosis and Haemostasis (ISTH) overt DIC criteria. The serum levels of angiogenic factors were measured at the time of admission (Day 1), Day 3 and Day 5. This study compared levels of these angiogenic factors between the two DIC groups, and evaluated their predictive value for organ dysfunction.

**Results:**

DIC patients, especially those with ISTH DIC, showed higher Sequential Organ Failure Assessment (SOFA) scores and lactate levels. There were lower levels of VEGF, Ang1 and the soluble Tie2 in the ISTH DIC patients than the non-DIC patients. The levels of soluble VEGF receptor-1 (sVEGFR1), Ang2 and the Ang2/Ang1 ratio in the ISTH DIC patients were higher than in non-DIC patients. The relationship between the presence of massive transfusion and angiogenic factors indicated the same results. The levels of sVEGFR1, Ang2 and the Ang2/Ang1 ratio correlated with the SOFA scores. In particular, sVEGFR1 and Ang2 were independent predictors of an increase in the SOFA score. The lactate levels independently predicted increases in the levels of sVEGFR1 and Ang2. The decrease in the platelet counts also independently predicted the increase in Ang2 levels in DIC patients.

**Conclusions:**

Angiogenic factors and their soluble receptors, particularly sVEGFR1 and Ang2, are considered to play pivotal roles in the development of organ dysfunction in DIC associated with severe trauma. DIC-induced tissue hypoxia and platelet consumption may play crucial roles in inducing sVEGFR1 and Ang2, and in determining the prognosis of the severity of organ dysfunction.

## Introduction

The survival of trauma patients depends on the ability to control two opposing conditions, namely, bleeding during the early phase and thrombosis during the late phase of trauma [1]. Hess *et al. *[2] suggested a new concept regarding the pathophysiological mechanism of trauma-related hemostatic changes, Acute Coagulopathy of Trauma-Shock (ACoTS). However, we have previously demonstrated that this could be best explained by the development of disseminated intravascular coagulation (DIC) [1,3].

While the activation of the tissue-factor-dependent pathway at the top of the cascade is the same, DIC is subdivided into a fibrinolytic and thrombotic phenotype [3]. DIC at an early phase of trauma, approximately 24 to 48 hours after injury, shows the fibrinolytic phenotype which affects the patient prognosis due to massive bleeding [1,4]. DIC at a late phase of trauma is a thrombotic phenotype which also affects the patient prognosis due to the development of multiple organ dysfunction syndrome (MODS) [1,3,5].

Vascular endothelial growth factor (VEGF) plays crucial roles in angiogenesis and microvascular permeability [6]. VEGF mainly binds to two transmembrane receptors, VEGF receptor-1 (VEGFR1) and VEGFR2. VEGFR2 mainly mediates endothelial growth, survival signals and pathological angiogenesis. In contrast, VEGFR1-mediated signaling plays important roles by increasing the vascular permeability under pathological conditions, such as ischemia and inflammation.

The angiopoietin (Ang)-Tie2 ligand-receptor system is restricted to regulation of the endothelium and it is also involved in multiple organ dysfunction-related pathways [7]. The Ang-Tie2 system not only regulates angiogenesis, but also controls endothelial inflammation, along with VEGF and its receptor system [8]. Ang1 stabilizes the endothelial cells, inhibits vascular leakage, and suppresses inflammatory and coagulation-related gene expression through Tie2 activation [8-10]. Ang2 antagonizes the binding of Ang1 to Tie2. Therefore, Ang2 is thought to act as a proinflammatory mediator increasing fluid leakage through the endothelial vasculature [11]. Several studies have demonstrated that the ratio between Ang1 to Ang2 better describes the state of activation of the endothelium, because Ang1 and Ang2 have agonist-antagonist properties on the endothelium [12,13].

Clinical studies have demonstrated the serum levels of VEGF to be strongly enhanced after polytrauma and burn injuries [14]. Ang2 plays a critical role in the pathogenesis of the activated endothelium with increased vascular permeability observed early after trauma [12]. Moreover, the Ang2 levels in multi-trauma patients are increased upon the development of septic complications connected with a poor prognosis [15]. However, no previous reports have documented the data regarding these angiogenic factors and their soluble receptors in DIC associated with severe trauma. This study tested the hypothesis that the angiogenic factors play pivotal roles in the development of DIC and DIC-induced organ dysfunction associated with severe trauma. The present study examined the serial changes in serum angiogenic factors and their soluble receptors in patients with trauma-associated DIC and investigated the relationships between these factors, receptors and organ dysfunction.

## Materials and methods

### Patients

With the approval of the Institutional Review Board and after written informed consent was obtained from either the patients or their kin, severe trauma patients were enrolled in the study. Severe trauma patients were defined as those with an Injury Severity Score (ISS) ≥ 9 (at least one abbreviated Injury Scale ≥ 3) [16]. Patients were excluded if they were under 18 years of age, had a terminal illness, were receiving anticoagulant therapy, had known clotting disorders, such as hematopoietic malignancies or severe liver cirrhosis, or were complicated with cardiac arrest. No patients received anticoagulant drugs, such as heparin, in consideration of re-bleeding during the study period. In addition, no antifibrinolytic drugs, such as tranexamic acid, were administered because the blood samples and patients' information for this study were collected before publication of another study showing the importance of early treatment with tranexamic acid in bleeding trauma patients [17]. Fifteen healthy volunteers served as the control subjects. The patient source was the same as that described in our previous study [18].

### Definitions

The severity of illness of the patients was evaluated according to the Acute Physiology and Chronic Health Evaluation (APACHE) II score at the time of enrollment. Organ dysfunction was assessed by the Sequential Organ Failure Assessment (SOFA) score within 24 hr after arrival at the emergency department (Day 1), as well as on Days 3 and 5. The DIC diagnosis was made on the Japanese Association for Acute Medicine (JAAM) DIC diagnostic criteria within five days after injury [19]. The overt DIC scores based on the International Society on Thrombosis and Hemostasis (ISTH) were also calculated [20]. The fibrin/fibrinogen degradation product (FDP) was used as the fibrin-related marker in the ISTH criteria. No increase, a moderate increase and a strong increase were defined as a FDP of less than 9, 10 to 24 and more than 25 mg/L, respectively. When the total score was ≥ 5, a diagnosis was established by the ISTH criteria. Patients that met the DIC criteria during the study period were defined as DIC patients. A massive transfusion was defined as a transfusion of more than 10 U (1,400 ml) of packed red blood cells. The main outcome measure was the 28-day mortality.

### Study protocol and measurement methods

Blood samples were collected by an arterial catheter within 12 hours after arrival at the emergency department (Day 1), as well as on Days 3 and 5. The blood was immediately placed into individual tubes and centrifuged at 3,000 rpm, for five minutes at 4°C. The serum and/or plasma were stored at -80°C until used for the assay. Serum samples were used for all measurements.

The following variables were measured in duplicate: VEGF (Quantikene; Human VEGF; R&D Systems, Inc., Minneapolis, MN, USA); sVEGFR1 (Quantikene; Human sVEGF R1/Flt-1; R&D Systems, Inc.), sVEGFR2 (Quantikene; Human sVEGF R2/KDR/Flk-1; R&D Systems, Inc.); Ang1 (Quantikene; Human Angiopoietin-1; R&D Systems, Inc.); Ang2 (Quantikene; Human Angiopoietin-2; R&D Systems, Inc.); and soluble Tie2 receptor (sTie2) (Quantikene; Human Tie-2; R&D Systems, Inc.). The platelet count, prothrombin time, fibrinogen level, FDP, D-dimer, and antithrombin were also measured in the patient plasma to establish the diagnosis and determine the treatment of DIC. In addition, the ABL SYSTEM 620 (Radiometer, Copenhagen, Denmark) was used for the lactate measurement.

A sufficient amount of platelet concentration, fresh frozen plasma (FFP), and packed red blood cells (PRBC) were transfused, based on repeatedly obtained laboratory data to maintain the hemodynamics and to treat hemostatic disorders.

### Statistical analysis

The statistical analyses and calculations were performed with the SPSS 19.0 software package (SPSS, Inc., Chicago, IL, USA). Differences between the two groups were analyzed using a two-sided nonparametric Mann-Whitney U test, and categorical variables were compared using Pearson's chi-square test or Fisher's exact test when required. The Kruskal-Wallis analysis of variance was used to compare the three groups. Correlations were evaluated by the Spearman's rank test. Multiple regression analyses were undertaken to determine contributions to SOFA score. The relationship between the dependent and the independent variables were analyzed by a multiple regression analysis using the stepwise method. The results of the regressions were reported as the partial regression coefficient (B), 95% confidence intervals (CI). The coefficient of determinant (*R*^2^) was also reported. A *P-*value < 0.05 was considered to be statistically significant. Unless otherwise stated, all results were expressed as the means ± SEM.

## Results

### Patients' characteristics

Fifty-seven patients with severe trauma met the inclusion criteria and were eligible for the present study. Patients were subdivided into a DIC group (*n *= 30) or a non-DIC group (*n *= 27), based on the JAAM DIC criteria. The DIC patients were further subdivided into those who met only the JAAM DIC criteria (JAAM DIC; *n *= 21) and those who simultaneously met both the JAAM and ISTH overt DIC criteria (ISTH overt DIC; *n *= 9). The baseline characteristics of the patients are shown in Table 1. The DIC group had a significantly higher maximal SOFA score (*P *< 0.001), although the ISS, APACHE II scores, P/F ratio and the prevalence of acute respiratory distress syndrome (ARDS) and sepsis were not significantly different between the DIC and non-DIC groups (ISS, *P *= 0.394; APACH II score, *P *= 0.277; P/F ratio, *P *= 0.194; ARDS, *P *= 0.823; sepsis, *P *= 0.813). The DIC groups received significantly more blood products (packed red blood cell, *P *= 0.001, FFP, *P *= 0.004, platelet concentrate, *P *= 0.032). Table 2 shows the Day 1 data of platelet counts, coagulation and fibrinolysis factors, and lactate levels. The higher FDP and D-dimer levels in DIC patients suggested disseminated fibrin thrombosis.

**Table 1:** The baseline clinical characteristics of the patients

		DIC		*P *value
			
	Non-DIC	JAAM DIC	ISTH overt DIC	
	**(*n *= 27)**	**(*n *= 21)**	**(*n *= 9)**	
Age (years)	50.0 ± 3.7	43.9 ± 4.5	57.2 ± 7.6	0.273
Gender (male/female)	19/8	10/11	4/5	0.192
ISS	26.0 ± 2.7	27.8 ± 2.3	31.7 ± 4.9	0.394
APACHE II score	19.2 ± 1.3	18.0 ± 1.1	24.4 ± 3.6	0.277
SOFA score max	4.5 ± 0.4	5.9 ± 0.5	9.8 ± 1.3	< 0.001
Sepsis (yes/no)	2/25	1/20	1/8	0.813
P/F ratio min	197 ± 21	185 ± 24	134 ± 35	0.194
ARDS (yes/no)	14/13	11/10	6/3	0.823
JAAM DIC max	2.6 ± 0.3	4.9 ± 0.2	6.6 ± 0.44	< 0.001
ISTH DIC max	2.0 ± 0.1	3.4 ± 0.1	5.4 ± 0.2	< 0.001
Packed red blood cell (ml)	491 ± 144	914 ± 174	2436 ± 594	0.001
Massive transfusion (yes/no)	3/24	5/16	5/4	0.026
FFP (ml)	445 ± 148	1032 ± 429	2503 ± 1011	0.004
Platelet concentrate (U)	3.5 ± 8.9	7.4 ± 3.2	26.7 ± 12.4	0.032
Outcome (survived/died)	26/1	20/1	6/3	0.029

DIC, disseminated intravascular coagulation; ISS, Injury Severity Score; APACHE, Acute Physiology and Chronic Health Evaluation; SOFA, Sequential Organ Failure Assessment; MODS, multiple organ dysfunction syndrome; JAAM, Japan Association for Acute Medicine; ISTH, International Society on Thrombosis and Haemostasis; FFP, fresh frozen plasma. We defined the maximum or minimum score as the highest or lowest score on day1, day3 and day5, when we collected the patient data and samples. Packed red blood cells, FFP, and Platelet concentrate indicate the total volume within 5 days after injury.

**Table 2:** The platelet counts, coagulation and fibrinolysis factors, and lactate levels on day1

		DIC		*p *value
			
	Non-DIC	JAAM DIC	ISTH overt DIC	
Platelet counts (10^9^/L)	153.4 ± 12.3	141.1 ± 11.9	110.0 ± 27.0	0.295
Prothrombin time (sec)	13.0 ± 0.3	13.7 ± 0.3	17.9 ± 1.3	< 0.001
Fibrinogen (g/L)	2.46 ± 0.19	1.88 ± 0.13	1.04 ± 0.06	< 0.001
FDP (mg/L)	19.1 ± 4.0	55.0 ± 7.9	150.6 ± 55.6	< 0.001
D-dimer (μg/mL)	30.6 ± 10.5	32.4 ± 5.6	79.7 ± 21.8	0.004
Antithrombin (%)	77.8 ± 3.5	71.7 ± 3.3	59.0 ± 8.0	0.014
Lactate (mmol/L)	2.9 ± 0.3	4.3 ± 0.7	6.8 ± 1.8	0.010

The abbreviations are same as Table 1. FDP, fibrin/fibrinogen degradation products.

### Serial changes in angiogenic factors and their soluble receptors

**Figure 1:**
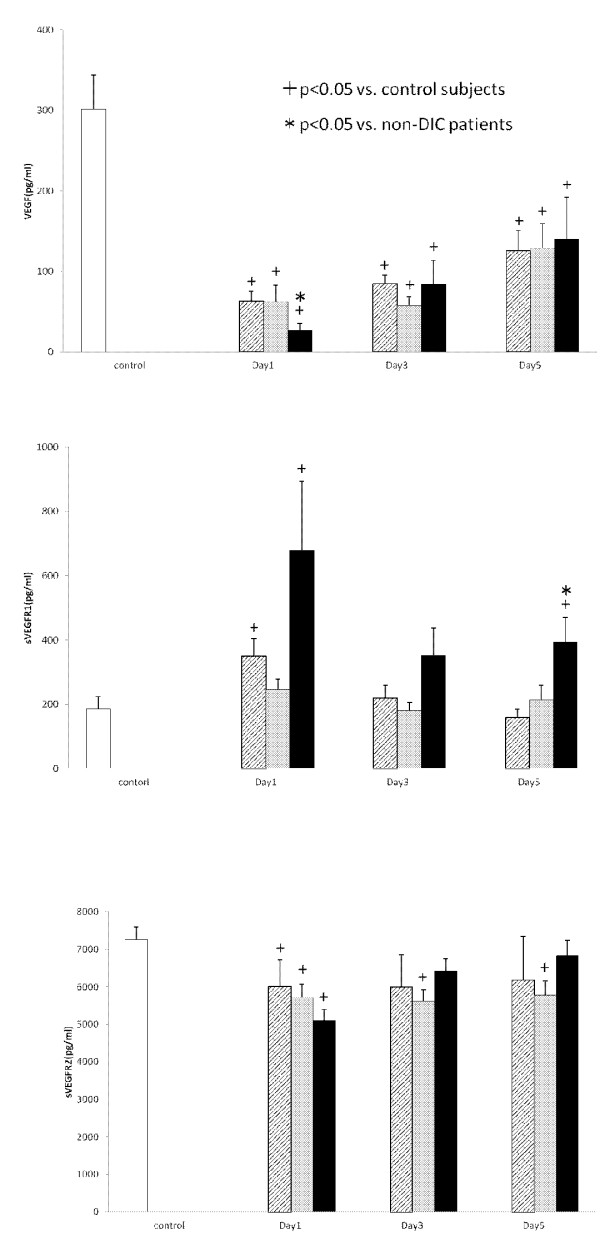
**The levels of VEGF, sVEGFR1 and sVEGFR2 in the serum**. White bars, control subjects; Hatched bars, non-DIC patients; Gray bars, JAAM DIC patients; Black bars, ISTH DIC patients.

**Figure 2:**
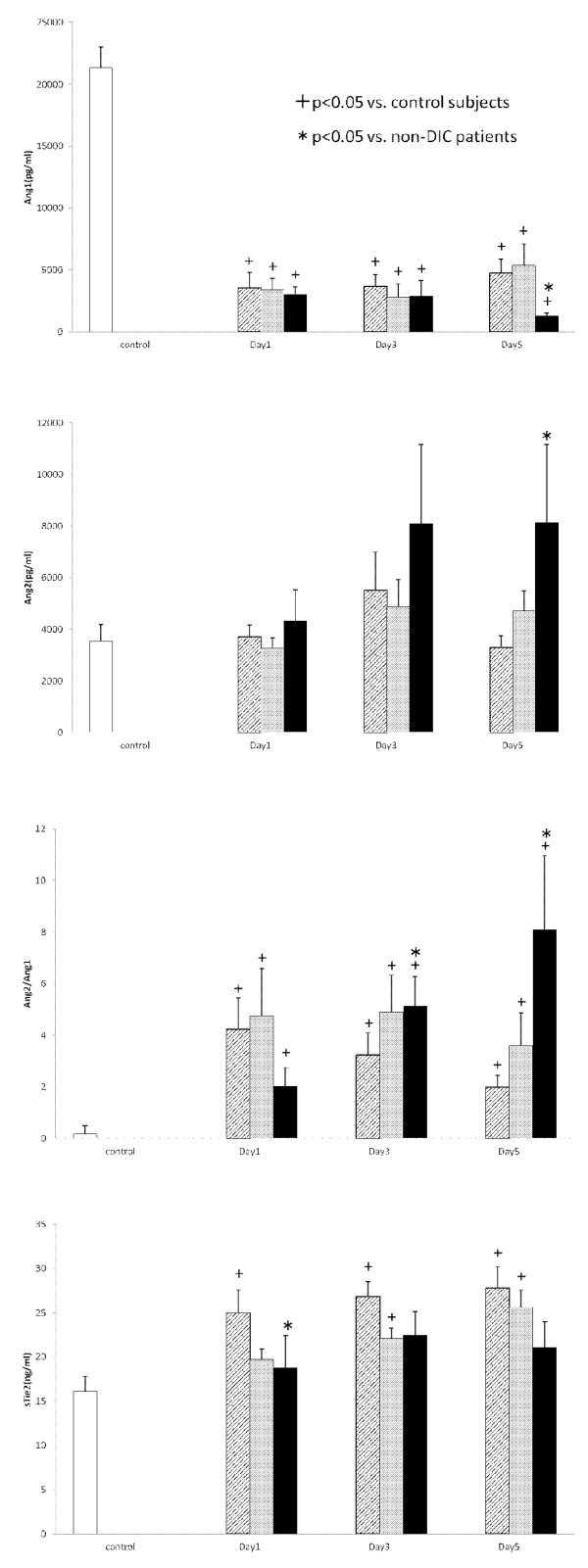
**The levels of Ang1, Ang2, sTie2 and the ratio of Ang2/Ang1 in the serum**. White bars, control subjects; Hatched bars, non-DIC patients; Gray bars, JAAM DIC patients; Black bars, ISTH DIC patients.

**Figure 3:**
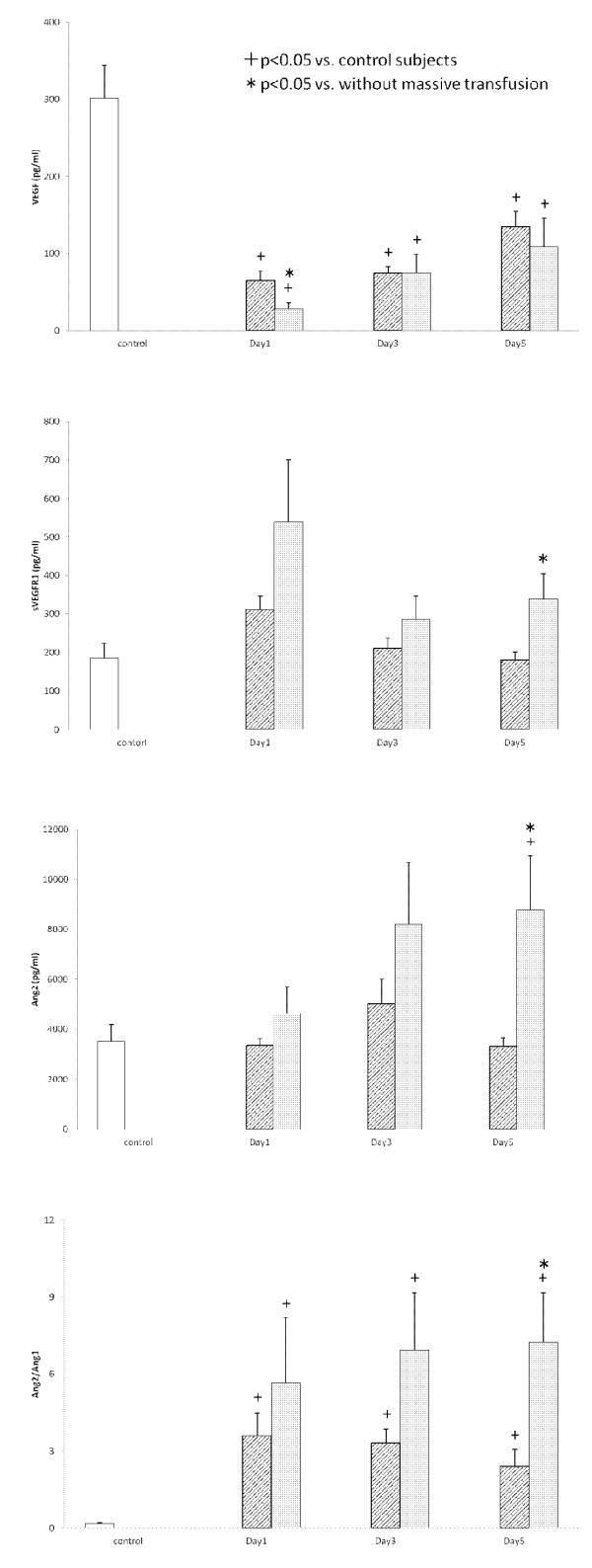
**The levels of VEGF, sVEGFR1, Ang2 and the ratio of Ang2/Ang1 in the serum**. White bars, control subjects; Hatched bars, without massive transfusion group; Gray bars, with massive transfusion group.

Serial changes in the circulating VEGF, sVEGFR1 and sVEGFR2 levels are presented in Figure 1. All three groups showed significantly lower levels of VEGF in comparison to the controls. In particular, those with the ISTH overt DIC group showed significantly lower VEGF levels on Day 1 in comparison to non-DIC subjects (*P *= 0.036). Although the sVEGFR1 levels on Days 1 and 3 did not differ between non-DIC and DIC groups, the sVEGFR1 levels on Day 5 in the ISTH overt DIC group were significantly higher than those of the non-DIC group (*P *= 0.004). On the other hand, the sVEGFR2 levels were not significantly different between the two groups during the study period. Figure 2 presents the Ang1, Ang2 and sTie2 levels and the Ang2/Ang1 ratio. This figure shows that there were significantly lower levels of Ang1, and higher levels of Ang2 in the ISTH overt DIC on Day 5 (Ang1, *P *= 0.026; Ang2, *P *= 0.038). Therefore, the ratio of Ang2 to Ang1 on Day 5 significantly increased in the ISTH overt DIC (*P *= 0.005). In addition, the sTie2 levels on Day 1 were lower in the ISTH overt DIC than those in the non-DIC group (*P *= 0.010). The patients were subdivided into with and without a massive transfusion. Figure 3 demonstrates the relationships among angiogenic factors, their soluble receptors and the presence of massive transfusion. There were significantly lower levels of VEGF on Day1, and higher levels of sVEGFR1 on Day 5 in the massive transfusion group (VEGF on Day 1, *P *= 0.022; sVEGFR1 on Day 5, *P *= 0.027). Moreover, the Ang2 and the Ang2/Ang1 ratio on Day 5 significantly increased in the massive transfusion group (Ang2 on Day 5, *P *< 0.001, Ang2/Ang1 on Day 5, *P *< 0.001). Other angiogenic factors and their receptors showed no statistical difference during the study period.

### Relationships between angiogenic factors, their soluble receptors and organ dysfunction

Table 3 shows that there were significant positive correlations between the sVEGFR1, Ang2, Ang2/Ang1 and the SOFA scores in DIC patients. A multiple regression analysis suggested that sVEGFR1 and Ang2 are independent predictors of an increase in the SOFA scores in DIC patients (Table 4).

**Table 3:** Spearman's correlation between the angiogenic factors, and the SOFA scores in DIC patients

	Rho	*P *value
SOFA-VEGF	-0.073	0.514
SOFA-sVEGFR1	0.253	0.022
SOFA-sVEGFR2	-0.062	0.580
SOFA-Ang1	0.043	0.699
SOFA-Ang2	0.389	< 0.001
SOFA-Ang2/Ang1	0.233	0.035
SOFA-sTie2	0.017	0.882

**Table 4:** The results of the multiple regression analysis using a stepwise method for prediction of the maximum of SOFA score in DIC patients

	B	SE	β	*P *value	95% CI
sVEGFR1 max	0.004	0.001	0.460	0.007	0.001-0.006
Ang2 max	0.000	0.000	0.411	0.014	0.000-0.000

*R*^2 ^= 0.630, ANOVA *P *< 0.001

B, partial regression coefficient; SE, standard error; ß, standard partial regression coefficient; CI, confidence interval; *R*^2^, coefficient of determinant; ANOVA, analysis of variance. Peak and nadir values of all angiogenic factors and their soluble receptors, were defined respectively as the maximal and minimal levels measured for each patient during the course of data collection. Potential predictor variables included the presence of massive transfusion, all peak and nadir values of all angiogenic factors and their receptors.

### Factors influencing the changes in angiogenic factors and their soluble receptors

A multiple regression analysis was performed to determine the contributions to sVEGFR1 and Ang2 changes in DIC patients. Table 5 shows that the maximal lactate level is an independent predictor of changes in the sVEGFR1 and Ang2 expression in the DIC patients. Minimal platelet counts also independently predicted the increase in the sVEGFR1 levels in patients with DIC.

**Table 5:** The result of the multiple regression analysis with stepwise method for prediction of sVEGFR1 and Ang2 in DIC patients

	B	SE	β	*P *value	95% CI
sVEGFR1					
Lactate max	49.1	15.8	.464	0.004	16.65-81.52
Platelet min	-33.2	13.4	-0.372	0.019	-60.6- -5.794
Ang2					
Lactate max	1080.2	218.7	0.682	< 0.001	632.2-1528.1

sVEGFR1; *R*^2 ^= 0.491, ANOVA *P *< 0.001

Ang2; *R*^2 ^= 0.466, ANOVA *P *< 0.001

The abbreviations are same as Table 4.

Potential predictor variables included the peak prothrombin time, FDP, D-dimer, and lactate levels and nadir platelet counts, fibrinogen and antithrombin during the course of data collection.

## Discussion

The present study has demonstrated that severe trauma patients with associated DIC showed significant decreases in their VEGF, Ang1, and sTie2 levels, and increases in their SOFA scores, sVEGFR1 and Ang2 levels as well as in the Ang2/Ang1 ratio. sVEGFR1 and Ang2 were the most prominent predictors of increases in the SOFA scores in the DIC patients. We also observed lower levels of VEGF, and higher levels of sVEGFR1 in the massive transfusion group. In addition, the Ang2, as well as the Ang2/Ang1 ratio, significantly increased in the massive transfusion group. However, massive transfusion was not found to be an independent factor for predicting the SOFA scores in the multivariable analysis.

Previous studies have demonstrated the JAAM DIC diagnostic criteria to have an acceptable validity for the diagnosis of DIC at an early phase of trauma, and JAAM DIC exists along with a continuum to ISTH overt DIC [4,21]. In addition, we previously reported that DIC at an early phase of trauma, which was based on the JAAM DIC criteria and ISTH DIC criteria, continuously progressed to DIC at a late phase until five or six days after the trauma [22].

Major trauma causes massive tissue injury, and initiates a systemic inflammatory response syndrome. Endothelial activation and dysfunction play a major role in the development of organ injuries after trauma [23]. Angiogenic factors, such as VEGF, angiopoietin and their receptors, have complex effects on endothelial cells [6,7].

Grad *et al. *[14] demonstrated that the serum VEGF levels in trauma and burn patients with complications, such as sepsis, ARDS or multiple organ dysfunction syndrome (MODS), are lower than those of the patients without such complications. Lower VEGF levels are also associated with organ dysfunction and a poor outcome in patients with sepsis [24,25]. The current results were consistent with these studies. However, several studies have reported that the plasma VEGF levels in the patients with septic shock are higher than those of patients without shock, and that the VEGF concentration at the time of admission correlates with the severity of disease [26,27]. Therefore, the levels of VEGF in patients with sepsis, septic shock and other critical illnesses remain controversial [12,28]. The present study measured the VEGF levels using serum samples. Webb *et al. *[29] suggested that plasma is the preferred medium to measure VEGF because the platelet-mediated secretion of VEGF during the clotting process could artificially increase the levels of VEGF. Furthermore, this study indicated that the coexistence of sVEGFR1 reduces the detection of VEGF. Platelets are one of the main transporters of circulating VEGF, and thrombin-activated platelets release VEGF [30,31]. We observed a significant correlation between the platelet counts and VEGF levels, and marked increases in the levels of sVEGFR1 in DIC patients with sepsis (our unpublished data; S Jesmin, T Wada, S Gando, *et al*.). Therefore, we believe that the decrease in the platelet counts and the elevation of sVEGFR1 are the main causes of lower VEGF levels. We showed a significant correlation between the VEGF and sVEGFR1 levels (Spearman rho = -0.236, *P *= 0.003, *n *= 150), but no correlation was observed between the platelet counts and VEGF in the present study. The precise mechanism of lower VEGF levels remains unclear; however, we speculate that the increase in the sVEGFR1 levels may be one of the causes of the lower VEGF levels in severe trauma patients, especially in those with DIC.

The present study found the sVEGFR1 levels in the ISTH overt DIC patients to be significantly higher than those of the non-DIC patients on Day 5 and sVEGFR1 was an independent predictor of the increase in the SOFA scores in patients with DIC associated with severe trauma. sVEGFR1 is generated by alternative splicing of VEGFR1 mRNA and functions as a decoy molecule, competing with VEGFR1 for binding to VEGF [27]. VEGF and VEGFR1 on the cell surface are up-regulated after endothelial injury, promoting re-endothelialization by the enhancement of vascular remodeling [32]. The results suggest that higher sVEGFR1 levels may hamper VEGF signaling in the process of endothelial repair, thereby leading to disease progression and organ dysfunction [28,33]. Furthermore, high circulating sVRGFR1 levels have been reported to correlate with morbidity and mortality, and are a potent marker of disease severity in septic or critically ill patients [27,28,34]. The current results were in agreement with these previous studies. On the other hand, an *in vivo *study suggested that VEGFR1 is involved in the migration of monocytes/macrophages, and that elevation of sVEGFR1 leads to an anti-inflammatory state [28,35]. The present results and the previous studies indicate that sVEGFR1 may have dual roles in the inflammatory processes, being involved in both endothelial cell damage and in inducing an anti-inflammatory effect. However, the inflammation caused by the high sVEGFR1 level may overcome its anti-inflammatory effects, leading to organ dysfunction in patients with DIC associated with trauma.

sVRGFR2 is a soluble truncated form of VEGFR2. Like sVEGFR1, sVEGFR2 may have regulatory consequences with respect to VEGF-mediated angiogenesis. However, its precise role has not yet been clarified [36]. The present study found no significant differences in the levels of sVEGFR2 between the DIC group and non-DIC group, thus suggesting that it does not have a major role in DIC after severe trauma.

Previous studies indicated that the serum levels of Ang2 are related to poor prognosis, and are independently correlated with the severity of injury and tissue hypoperfusion in multi-trauma patients [12,15]. Other studies also show that lower Ang1 and higher Ang2 levels are associated with a poor outcome in patients with sepsis or critical illness [9,24,37,38]. Ang1 has anti-inflammatory properties and protects against vascular leakage, while Ang2 promotes inflammation and increases the vascular permeability, leading to the development of ARDS [8,9,39,40]. Moreover, positive relationships between Ang2 and inflammatory cytokines, such as TNF-alpha and IL-6, have been observed [37]. These results suggest that elevations in Ang2 and a decrease in Ang1 may reflect a pro-inflammatory state, and that this is best summarized by the ratio between Ang1 and Ang2 [12,13]. The current study and previous studies suggest that a higher Ang2 level, as well as an imbalance of Ang1 and Ang2 (high Ang2/Ang1 ratio), are both associated with inflammation during DIC associated with severe trauma, thus resulting in organ dysfunction. We observed that DIC associated with sepsis has lower levels of Ang1 and higher levels of Ang2 on Day 1 (our unpublished data; S Jesmin, T Wada, S Gando, *et al*.). The present study showed that the same changes could be found on Day 5, when the fibrinolytic DIC proceeded into thrombotic DIC, which is accentuated in sepsis. These results suggest that the changes in the Ang levels may, therefore, play some role in the development of thrombotic DIC. Furthermore, the administration of Ang1 protects the vasculature from leakage, countering the potentially lethal actions of VEGF and inflammatory agents in animal experiment [41]. The correction of the imbalance between Ang1 and Ang2 by administration of Ang1 or inhibition of Ang2 may represent a new therapeutic target for severe inflammatory illnesses, such as DIC related to trauma.

Soluble Tie2 (sTie2) is released by proteolytic cleavage of the extracellular domain by matrix metalloproteases, which occurs constitutively or after VEGF signaling [42]. Cleaved sTie2 binds to both Ang1 and Ang2 to inhibit ligand-mediated Tie2 receptor activation and downstream signaling [43]. A previous study also showed that the sTie2 levels correlate with the VEGF levels, supporting the *in vivo *shedding of Tie2 through VEGF signaling [44]. The sTie2 levels on Day 1, as well as the VEGF levels, were significantly lower in the ISTH overt DIC patients than in the non-DIC patients in the current series. This result coincided with those of previous studies. Yuan *et al. *[45] reported that Ang2 binds sTie2 with approximately 20-fold lower affinity than Ang1 does. Therefore, sTie2 may sift the Ang2/Ang1 in favor of Ang2. The present study found the sTie2 levels in ISTH DIC on Day 1 to be lower than those in non-DIC, but no significant difference was observed between the two groups on Day 5. The changes in the Ang1 and Ang2 levels in the ISTH DIC group on Day 5 may be affected by the augmentation of the sTie2 activity. These results and the previous studies may indicate that there is a close relationship between the VEGF/VEGFR signaling pathway and the Ang/Tie2 signaling system.

DIC patients in the current study showed higher levels of FDP, D-dimer and lactate, indicating tissue hypoxia induced by disseminated fibrin thrombosis. A multiple regression analysis also showed that lactate and platelet were independent predictors of an increase in the sVEGFR1 levels in DIC patients. DIC is characterized by the widespread activation of tissue factor, massive thrombin generation and fibrin formation, leading to microvascular occlusion and tissue hypoxia [46]. Any type of shock can precipitate DIC in response to tissue hypoxia, slow capillary flow, and endothelial damage [1]. Tissue hypoxia transcriptionally induces the expression of VEGFR1 [47]. In addition, a decrease in the platelet counts may correlate with the lower VEGF levels, leading to the elevation of sVEGFR1. These results indicate that DIC-induced tissue hypoxia and platelet consumption may play pivotal roles in inducing an alternatively spliced form of VEGFR1, sVEGFR1, in patients with DIC associated with severe trauma. The Ang2 levels of the DIC patients were also independently predicted by lactate. Inflammatory cytokines are thought to be stimuli for Ang2 production in the clinical setting [37]. The results of the current study suggest that DIC-induced tissue hypoxia is another major determination of circulating Ang2 in the same way as in the experimental study [48]. Therefore, DIC is considered to be closely involved in the pathogenesis of organ dysfunction after trauma. Further studies in a larger number of subjects in a more diverse population are thus needed to confirm the roles and clinical relevance of angiogenic factors, their receptors and the occurrence of DIC associated with severe trauma.

## Conclusions

This study demonstrated that there were lower serum levels of VEGF, Ang1, and higher sVEGFR1 and Ang2 levels, and an increased Ang2/Ang1 ratio in patients with ISTH overt DIC associated with severe trauma. We also observed lower levels of VEGF, and higher levels of sVEGFR1 in the massive transfusion group. In addition, the Ang2 as well as the Ang2/Ang1 ratio significantly increased in the massive transfusion group. The changes in sVEGFR1, Ang2 and the Ang2/Ang1 ratio changes were significantly correlated with the SOFA scores in DIC patients. In particular, sVEGFR1 and Ang2 were found to be independent predictors of an increase in the SOFA scores in DIC patients. Moreover, the maximum lactate levels and the minimum platelet counts independently predicted increases in the sVEGFR1 levels. These data, therefore, support the pathophysiological relationship between organ dysfunction in patients with DIC associated with severe trauma and angiogenic factors and their receptors.

## Key messages

• There are significant positive correlations among sVEGFR1, Ang2, Ang2/Ang1 ratio, and the SOFA scores in the trauma patients with DIC.

• sVEGFR1 and Ang2 are independent predictors of an increase in the SOFA scores in DIC trauma patients.

• The maximum lactate levels and the minimum platelet counts independently predicted increases in the sVEGFR1 levels.

• Lactate also independently predicts the Ang2 levels of the DIC patients.

• These changes are most prominent in patients with ISTH overt DIC.

## Abbreviations

ACoTS: Acute Coagulopathy of Trauma-Shock; Ang: angiopoietin; APACHE: Acute Physiology and Chronic Health Evaluation; ARDS: acute respiratory distress syndrome; DIC: disseminated intravascular coagulation; FDP: fibrin/fibrinogen degradation product; FFP: fresh frozen plasma; ISS: Injury Severity Score; ISTH: International Society on Thrombosis and Haemostasis; JAAM: Japan Association for Acute Medicine; MODS: multiple organ dysfunction syndrome; PRBC: packed red blood cells; SOFA: Sequential Organ Failure Assessment; VEGF: vascular endothelial growth factor; VEGFR: vascular endothelial growth factor receptor

## Competing interests

The authors declare that they have no competing interests.

## Authors' contributions

TW analyzed the results, drew the diagrams and wrote the manuscript. SJ had the initial idea, established the immunoassays, performed and supervised the experiments, and reviewed the manuscript. SG had the initial idea, designed and supervised the research, identified patients, collected samples, provided clinical data and reviewed the manuscript. SNS and SZ established the experiments. HY reviewed the manuscript. All authors read and approved the final manuscript.
